# Which Nursing Homes Received Paycheck Protection Program Funding?

**DOI:** 10.1002/alz.091671

**Published:** 2025-01-09

**Authors:** Norma Coe, Chuxuan Sun, Jasmine Travers, Kevin Chen, Robert Burke, Monika Pogorzelska‐Maziarz, Nicholas Castle, Jason Falvey

**Affiliations:** ^1^ University of Pennsylvania, Philadelphia, PA USA; ^2^ New York University, New York, NY USA; ^3^ Thomas Jefferson University, Philadelphia, PA USA; ^4^ West Virginia University, Morgantown, WV USA; ^5^ University of Maryland, Baltimore, MD USA

## Abstract

**Background:**

Nearly 90% of nursing homes in the US experienced severe staffing shortages during COVID‐19, which disproportionately affected over 50% of nursing home residents living with Alzheimer’s Disease and Related Dementia (ADRD). One major federal policy to support the maintenance of staffing levels during the COVID‐19 pandemic was the Paycheck Protection Program (PPP), a $75 billion program that provided loans to small businesses, including nursing homes. Loans were forgivable if at least 60 percent of the loan was spent on payroll providing an incentive to apply resources toward staffing. This paper examines the organizational and staffing characteristics of nursing homes that received PPP loans, including the prevalence of residents living with ADRD and Serious Mental Illness (SMI).

**Method:**

We examine a national cohort of non‐VA nursing homes eligible for PPP funding. We compare those who received a PPP loan to nursing homes that did not receive a PPP loan. PPP loan receipt was captured in the US Small Business Administration (SBA) PPP data from April 2020 to June 2021. We primarily use datasets from 2019 to examine baseline characteristics, including Nursing Home Compare data, the Minimum Dataset (MDS), LTC Focus, and the Payroll‐Based Journal data.

**Result:**

3,536 nursing homes received a PPP loan in any of the three rounds; 9,943 eligible nursing homes never received a PPP loan.

More than 50% of nursing home residents were living with ADRD, and nearly 1 in 3 had a serious mental illness. These rates were slightly higher among facilities receiving PPP loans.

Staffing in 2019 was inferior in nursing homes receiving PPP loans than those who did not on all measures: lower‐star ratings on both quality and staffing measures, higher turnover rates at the RN (9.7% vs. 9.0%), LPN (11.2% vs. 10.3%), and CNA levels (11.4% vs. 11.0%). COVID‐19 affected PPP‐receiving nursing homes more in terms of confirmed cases (522 vs. 511), resident deaths (107 vs. 97), and staffing shortages (22% vs. 18%).

**Conclusion:**

This first‐ever direct federal funding effort to support nursing home staff during a pandemic appears to have supported the most vulnerable nursing homes.

